# Correction: Intermittent fasting promotes type 3 innate lymphoid cells secreting IL-22 contributing to the beigeing of white adipose tissue

**DOI:** 10.7554/eLife.112592

**Published:** 2026-07-09

**Authors:** Hong Chen, Lijun Sun, Lu Feng, Xue Han, Yunhua Zhang, Wenbo Zhai, Zehe Zhang, Michael Mulholland, Weizhen Zhang, Yue Yin

**Keywords:** Mouse

Chen H, Sun L, Feng L, Han X, Zhang Y, Zhai W, Zhang Z, Mulholland M, Zhang W, Yin Y. 2024. Intermittent fasting promotes type 3 innate lymphoid cells secreting IL-22 contributing to the beigeing of white adipose tissue. *eLife* **12**:RP91060. doi: 10.7554/eLife.91060.

Published 27 March 2024

We were alerted via PubPeer to instances of image duplication in Figure 5G, Figure 1E, and Figure 1K. Upon further internal review of related panels, we also identified similar issues in the insets of Figure 5G and Figure 2G. We sincerely regret these errors, which we attribute to mistakes during the image selection process—specifically during the selection of raw files and the subsequent assembly of figures from original images. Upon thorough re-examination of our raw data and experimental records, we confirm that these issues resulted from human errors during image processing and figure assembly, rather than experimental fraud or intentional misconduct. These errors do not affect the overall conclusions of the study.

1. Figure 5G

This panel contained two errors: both the main panel and the inset for IL-22RKO-IF sWAT were duplicated (with rotation) from the WT-IF eWAT dataset. Our audit confirms that the eWAT histology images were correctly derived from the scanning files of WT-IF mice. The sWAT image in article for IL-22RKO-IF mice was mistakenly selected from the same group of pictures exported from the same raw scanning file of WT-IF eWAT section. Two different magnifications were selected as the main panel and inset. We have replaced the incorrect image with the authentic IL-22RKO-IF sWAT data. Re-analysis of the original tissue sections yielded consistent results, supporting our original conclusion: WT-IF mice exhibit typical beiging of sWAT under intermittent fasting, whereas knockout of IL-22R significantly attenuates the increment of multilocular lipid droplets and the decrement of adipocyte size in subcutaneous fat induced by intermittent fasting.

2. Figures 1E and 1K

The control flow cytometry scatter plots in Figures 1E and 1K were derived from the same mouse. While the control plot in Figure 1K is correct, the plot in Figure 1E was erroneously generated using an NCD control file from a different cohort (NCD/HFD/HFD-IF) during analysis with FlowJo. We have re-analyzed the raw files from the correct NCD and NCD-IF cohorts using FlowJo and have replaced the duplicated image in Figure 1E.

3. Figure 2G

The inset image for the PBS group is a legitimate intermediate magnification cropped from the same sWAT section and is correct. The eWAT inset was incorrectly inserted from an sWAT image due to a folder selection error. To eliminate any ambiguity, we have removed the erroneous inset in Figure 2G (and similarly in Figure 5G). Since these insets represented only local enlargements of the main images, their removal does not affect the scientific conclusions.

In support of these corrections, we shared the underlying, original raw images and data for these figures with the editorial office.

We are committed to maintaining the highest standards of research integrity and will submit a formal Correction to the journal to update these figures. We apologize for any confusion caused and thank the community for their vigilance.

The corrected Figure 5 (updated panel G IL-22RKO-IF sWAT and WT-IF eWAT images, and insets removed) is shown here:

**Figure 1:**
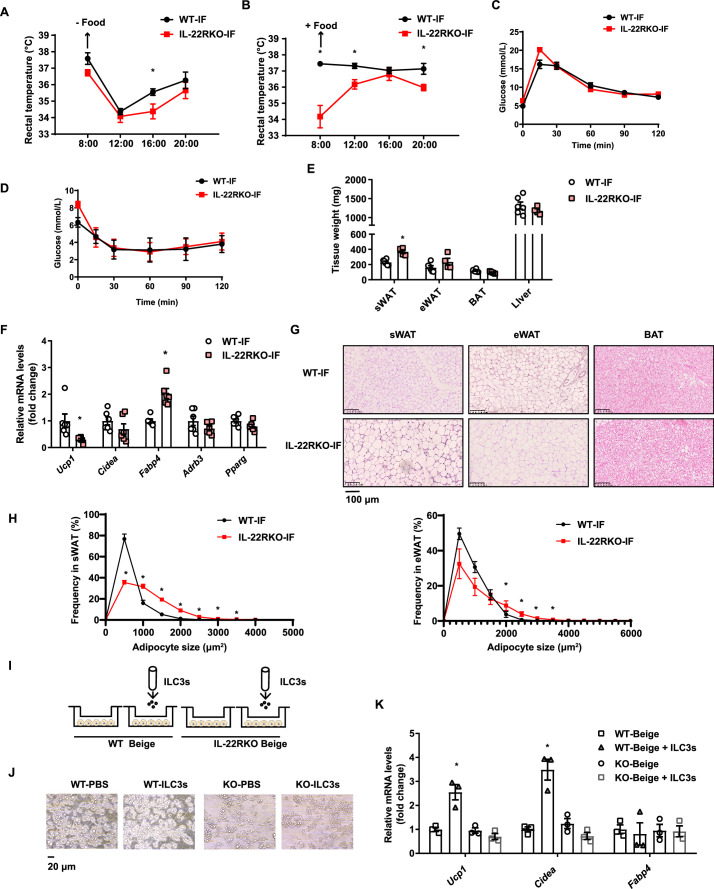


The originally published Figure 5 is shown for reference:

**Figure 2:**
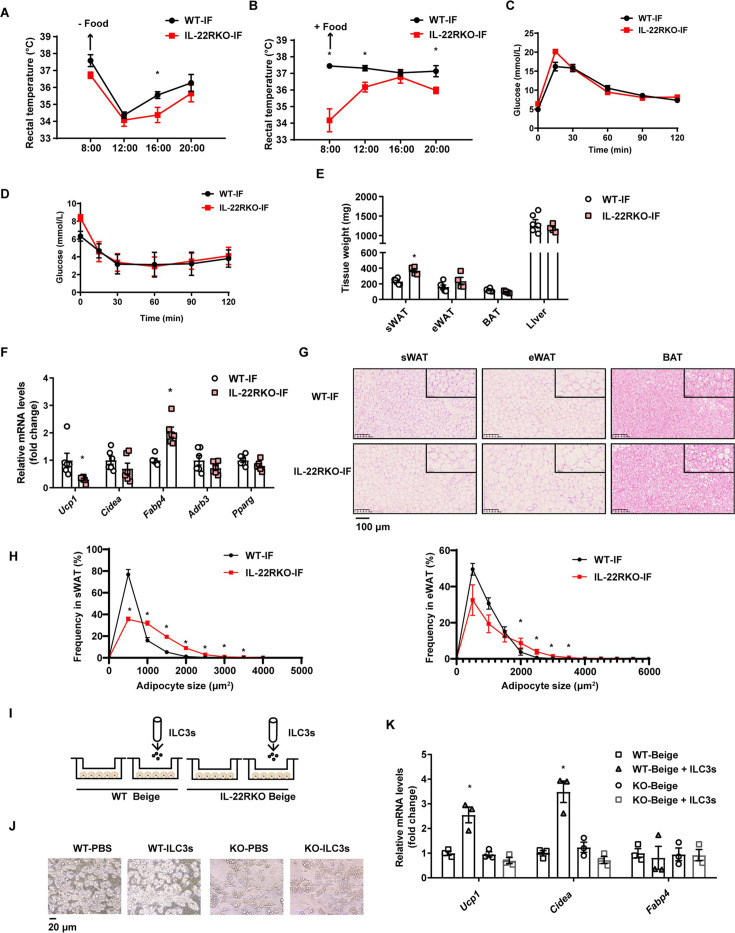


The corrected Figure 1 (updated for panel E NCD mice image and statistical graph) is shown here:

**Figure 3:**
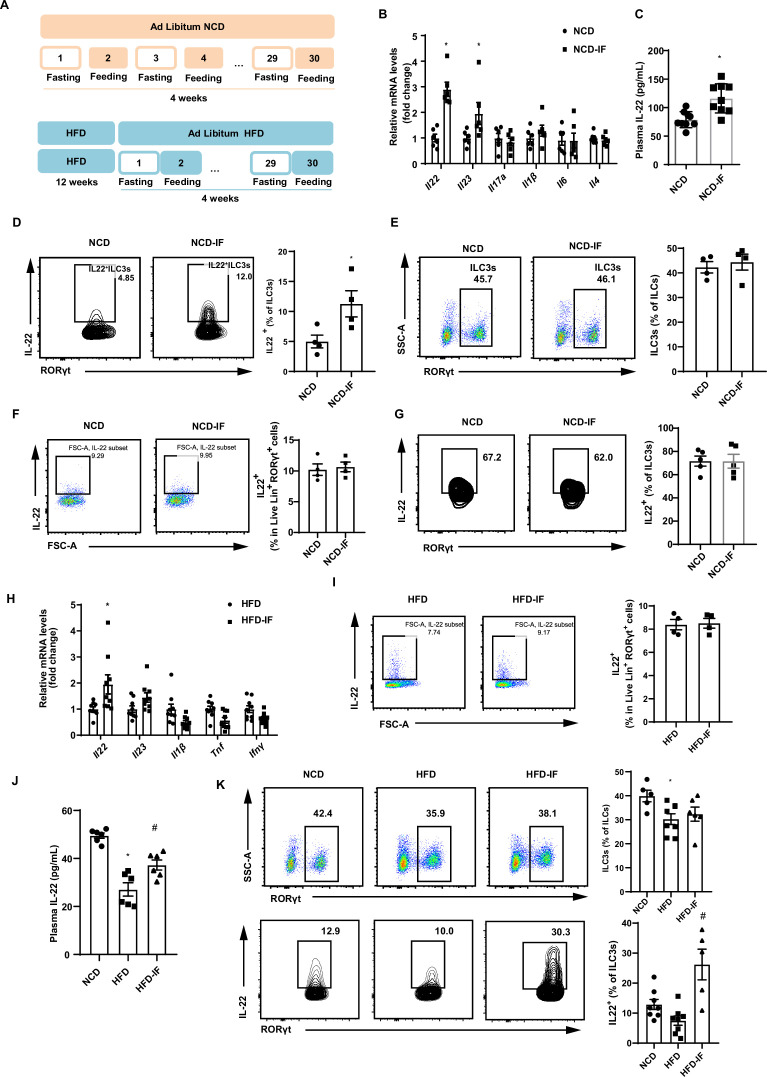


The originally published Figure 1G is shown for reference:

**Figure 4:**
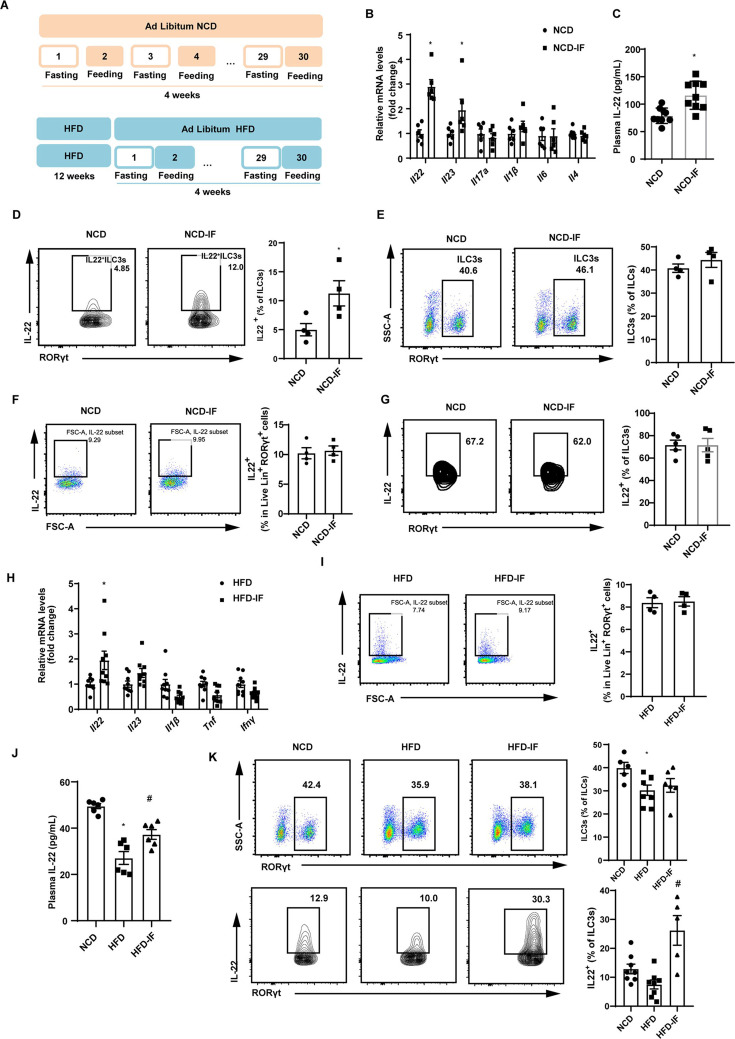


The corrected Figure 2 (updated panel G insets removed) is shown here:

**Figure 5:**
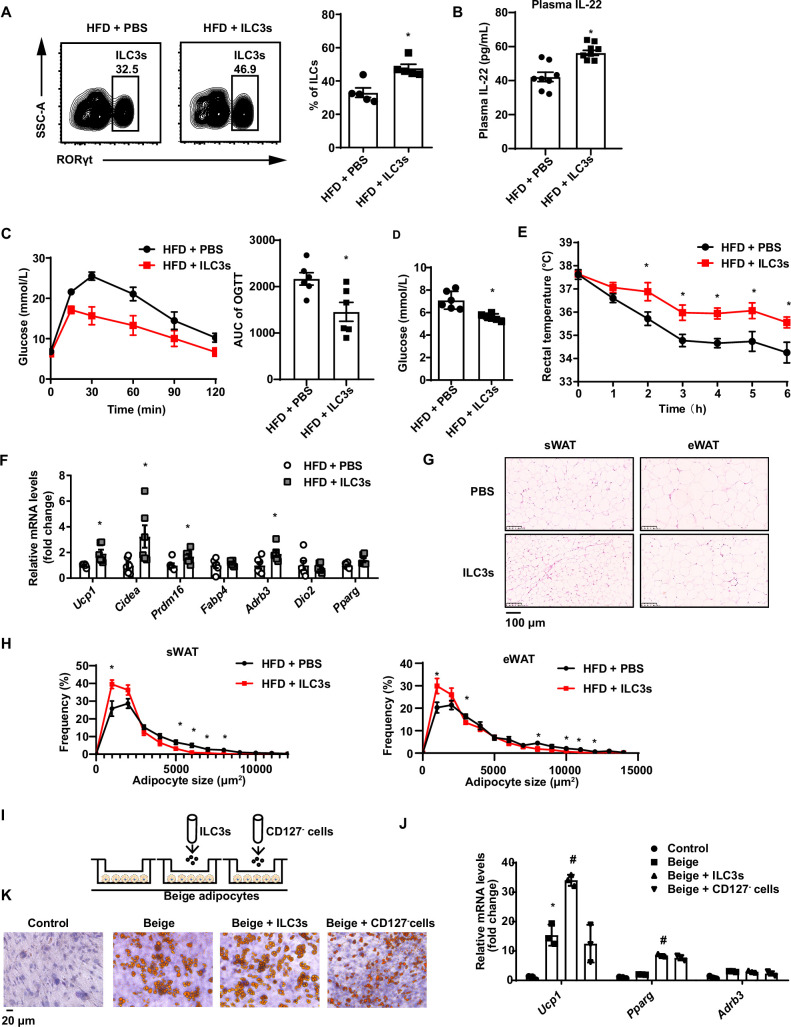


The originally published Figure 2 is shown for reference:

**Figure 6:**
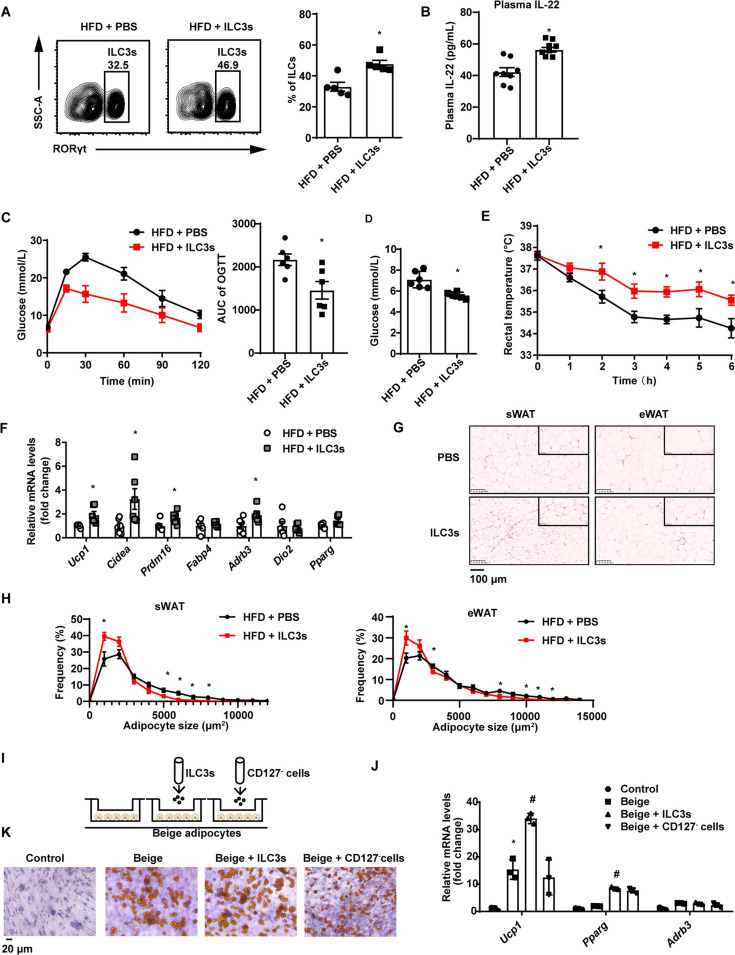


The article has been corrected accordingly.

