# Supplementary material for: Intermittent fasting promotes type 3 innate lymphoid cells secreting IL-22 contributing to the beigeing of white adipose tissue
Source: eLife. 2024 Mar 27;12:RP91060. doi: 10.7554/eLife.91060 (PMC10972562; doi:10.7554/eLife.91060)
Supplement: Supplementary file 1. [file elife-91060-supp1.docx]

**Supplementary File 1 Sequences of primers used in quantitative PCR.**

| ***Gene*** | **Forward Primer** | | **Reverse Primer** | |  |  |
| --- | --- | --- | --- | --- | --- | --- |
| *Il22* | ATGAGTTTTTCCCTTATGGGGAC | | GCTGGAAGTTGGACACCTCAA | |  |  |
| *Il17* | TCCCTCTGTGATCTGGGAA | | CTCGACCCTGAAAGTGAAGG | |  |  |
| *Il1β* | CCTTCCAGGATGAGGACATGA | | TGAGTCACAGAGGATGG-GCTC | |  |  |
| *Il6* | TAGTCCTTCCTACCCCAATTTCC | | TTGGTCCTTAGCCACTCCTTC | |  |  |
| *Ifnγ* | ATGAACGCTACACACTGCATC | | CCATCCTTTTGCCAGTTCCTC | |  |  |
| *Tbp* | ACCTTATGCTCAGGGCTTGG | | GCCGTAAGGCATCATTGGAC | |  |  |
| *RpL32* | GAGCAACAAGAAAACCAAGCA | | TGCACACAAGCCATCTACTCA | |  |  |
| *Hprt2* | TCAGTCAACGGGGGACATAAA | | GGGGCTGTACTGCTTAACCAG | |  |  |
| *Hsl* | CCAGCCTGAGGGCTTACTG | | CTCCATTGACTGTGACATCTCG | |  |  |
| *Adrb3* | GGCCCTCTCTAGTTCCCAG | | TAGCCATCAAACCTGTTGAGC | |  |  |
| *Chrebpβ* | AGATGGAGAACCGACGTATCA | | ACTGAGCGTGCTGACAAGTC | |  |  |
| *Dio2* | AATTATGCCTCGGAGAAGACCG | | GGCAGTTGCCTAGTGAAAGGT | |  |  |
| *Atp1a2* | CCACCACTGCGGAAAATGG | | GCCCTTAGACAGATCCACTTGG | |  |  |
| *Atgl* | AACACCAGCATCCAGTTCAA | | GGTTCAGTAGGCCATTCCTC | |  |  |
| *Fabp4* | | ACACCGAGATTTCCTTCAAACTG | | CCATCTAGGGTTATGATGCTCTTCA | |  |
| *Cidea* | | ATCACAACTGGCCTGGTTACG | | TACTACCCGGTGTCCATTTCT | |  |
| *Ppargc1α* | | GATTGAAGTGGTGTAGCGAC | | GTCGCTACACCACTTCAATC | |  |
| *Pparg* | | | TCAGCTCTGTGGACCTCTCC | | ACCCTTGCATCCTTCACAAG | |
| *Adrb3* | | GGCCCTCTCTAGTTCCCAG | | TAGCCATCAAACCTGTTGAGC | |  |
| *Prdm16* | | CAGCACGGTGAAGCCATTC | | GCGTGCATCCGCTTGTG | |  |
| *Il4* | | GGTCTCAACCCCCAGCTAGT | | GCCGATGATCTCTCTCAAGTGAT | |  |
| *Ucp1* | | CACCTTCCCGCTGGACACT | | CCCTAGGACACCTTTATACCTAATGG | |  |
| *Il23a* | | ATGCTGGATTGCAGAGCAGTA | | ACGGGGCACATTATTTTTAGTCT | |  |
| *Tnf* | | CGTCGTAGCAAACCACCAAG | | GAGATAGCAAATCGGCTGACG | |  |
| *Adrb2* | | GGGAACGACAGCGACTTCTT | | GCCAGGACGATAACCGACAT | |  |
| *Fgfr2* | | CCTCGATGTCGTTGAACGGTC | | CAGCATCCATCTCCGTCACA | |  |
| *Cd47* | | TGGTGGGAAACTACACTTGCG | | CGTGCGGTTTTTCAGCTCTAT | |  |
| *Cd274* | | GCTCCAAAGGACTTGTACGTG | | TGATCTGAAGGGCAGCATTTC | |  |
| *Slc7a1* | | CTGCCTCAACACCTATGACCT | | GAGAGCAGCAATCAAGAAGGAG | |  |
| *Ide* | | AATCCGGCCATCCAGAGAATA | | GGGTCTGACAGTGAACCTATGT | |  |
| *Cd74* | | AGTGCGACGAGAACGGTAAC | | CGTTGGGGAACACACACCA | |  |
| *Cd44* | | TCGATTTGAATGTAACCTGCCG | | CAGTCCGGGAGATACTGTAGC | |  |
| *Ccl4* | | TTCCTGCTGTTTCTCTTACACCT | | CTGTCTGCCTCTTTTGGTCAG | |  |
| *Il1b* | | GCAACTGTTCCTGAACTCAACT | | ATCTTTTGGGGTCCGTCAACT | |  |
| *Ccl3* | | TTCTCTGTACCATGACACTCTGC | | CGTGGAATCTTCCGGCTGTAG | |  |
| *Gata3* | | CTCGGCCATTCGTACATGGAA | | GGATACCTCTGCACCGTAGC | |  |
| *Rorγt* | | GACCCACACCTCACAAATTGA | | GACCCACACCTCACAAATTGA | |  |
